# Parental experiences of providing skin-to-skin care to their newborn infant—Part 1: A qualitative systematic review

**DOI:** 10.3402/qhw.v9.24906

**Published:** 2014-10-13

**Authors:** Agneta Anderzén-Carlsson, Zeni Carvalho Lamy, Mats Eriksson

**Affiliations:** 1 Centre for Health Care Sciences, Örebro University Hospital, Örebro, Sweden; 2 School of Health and Medical Sciences, Örebro University, Örebro, Sweden; 3 Departamento de Saúde Pública, Universidade Federal do Maranhão, Hospital Universitário, Sao Luis, MA, Brazil

**Keywords:** Kangaroo mother care, meta-study, newborn infant, skin-to-skin care, qualitative systematic review

## Abstract

**Aim:**

To describe parental experiences of providing skin-to-skin care (SSC) to their newborn infants.

**Background:**

SSC care for newborn infants has been reported to have positive physiological and psychological benefits to the infants and their parents. No systematic review regarding parental experiences has been identified.

**Design:**

In this first part of a meta-study, the findings of a systematic literature review on parental experience of SSC care are presented.

**Data sources:**

Four databases were searched, without year or language limitations, up until December 2013. Manual searches were performed in reference lists and in a bibliography of the topic.

**Review methods:**

After a quality-appraisal process, data from the original articles were extracted and analysed using qualitative content analysis.

**Results:**

The systematic and manual searches led to the inclusion of 29 original qualitative papers from nine countries, reporting experiences from 401 mothers and 94 fathers. Two themes that characterized the provision of SSC emerged: a restoring experience and an energy-draining experience.

**Conclusion:**

This review has added scientific and systematic knowledge about parental experiences of providing SSC. Further research about fathers’ experiences is recommended.

Skin-to-skin care (SSC) started as kangaroo mother care (KMC) in a Colombian hospital in the late 1970s as a way to avoid cross-infections caused by a shortage of incubators in neonatal units, which forced preterm and low-birth weight infants to share incubators with each other. Another aim of KMC was to facilitate contact between mothers and their newborn babies, thus preventing infant abandonment and humanizing neonatal care (Martinez, Rey, & Marquette, 1992). During SSC, the infant, dressed in only a diaper, lies on the parent's bare chest in an upright position for shorter or longer periods. A supportive binding or a special carrying pouch helps the parent to safely hold the baby close to the chest, where it can be breastfed in the kangaroo position (World Health Organisation, 2003).

Following the initial implementation of KMC in developing countries, several research projects focused on the physiological, psychosocial, and developmental effects of SSC. Most studies of physiological effects found that, compared to traditional neonatal care, SSC/KMC had the same or a better outcome. These studies focused on heart and breathing activity rates, variations and patterns, cerebral and body oxygenation, metabolism, temperature control and growth/weight gain (Charpak et al., 2005). Meta-analyses have revealed improvement in the following: mortality risk (Conde-Agudelo, Belizán, & Diaz-Rossello, 2011; Lawn, Mwansa-Kambafwile, Horta, Barros, & Cousens, 2010), nosocomial and severe infection/sepsis (Conde-Agudelo et al., 2011; Lawn et al., 2010), duration of hospital stay (Conde-Agudelo et al., 2011) and hypothermia (Conde-Agudelo et al., 2011; McCall, Alderdice, Halliday, Jenkins, & Vohra, 2010). KMC also led to increased weight, head circumference, and length (Conde-Agudelo et al., 2011). SSC has also been reported to reduce signs of procedural pain (Pillai Riddell et al., 2011).

Another research topic has been the impact of SSC/KMC on breastfeeding (Charpak et al., 2005). In infants with a low birth weight, meta-analyses demonstrated improved rates of breastfeeding and exclusive breastfeeding (Conde-Agudelo et al., 2011), while in healthy newborns breastfeeding frequency and duration were enhanced (Moore, Anderson Gene, Bergman, & Dowswell, 2012). Mothers in the SSC-groups breastfed exclusively to a greater extent at hospital discharge (Cattaneo et al., 1998; Marín Gabriel et al., 2010), a difference that in one study was lost at 1 month of age (Marín Gabriel et al., 2010) but in other lasted up to 3–6 months (Charpak, Ruiz-Pelaez, Figueroa de, & Charpak, 2001; Hake-Brooks & Anderson, 2008).

Skin-to-skin contact has also been found to have positive effects on psychosocial factors such as parental stress and mother–infant attachment/bonding and also on infant development (Charpak et al., 2005). In infants with low birth weight, a meta-analysis demonstrated better mother–infant attachment and interaction, parental and family satisfaction, and a better home environment (Conde-Agudelo et al., 2011).

A review by Moore et al. (2012) showed that mothers who held their infant in SSC showed less anxiety and more confidence about their abilities to take care of the infant after hospital discharge. The authors conclude that SSC improved early postpartum affectionate love/touch behaviour and also affectionate touch at 1 year (Moore et al., 2012). Some researchers have shown better moods in mothers in the SSC-group (De Macedo, Cruvinel, Lukasova, & D'Antino, 2007; Morelius, Theodorsson, & Nelson, 2005) and less depression (Bigelow, Power, Maclellan-Peters, Alex, & McDonald, 2012); a randomized controlled trial (RCT) from Spain could however not show any improvement in anxiety or depression (Marín Gabriel et al., 2010). SSC decreased stress measured with salivary-cortisol (Bigelow et al., 2012; Morelius et al., 2005) or self-rating (Tallandini & Scalembra, 2006). Improved infant development and mood has been shown in the SSC-group up to 6–12 months of age (Ohgi et al., 2002; Tessier et al., 2003).

Today, SSC/KMC is provided by parents within neonatal care worldwide, even in specialized high-tech neonatal units. The degree to which SSC/KMC is practiced varies from being used around the clock (continuous KMC, C-KMC) to shorter periods during the day (intermittent KMC, I-KMC) (Nyqvist et al., 2010). The use of SSC/KMC is recommended by the World Health Organization in maternity and special baby care settings (World Health Organisation, 2003). In the following, we will use the term SSC to include all provision of this method to newborn infants, regardless of gestation age of the infant, the duration of the SSC, and hospital setting.

As previously stated, meta-analyses have been conducted on physiological and psychosocial outcome (Conde-Agudelo et al., 2011; Lawn et al., 2010; McCall et al., 2010). The included studies on parental behaviour and mood were performed with parametric outcome measures such as self-rating or observational scales. Several studies using qualitative methodology have also explored experiences and perceptions of parents who participated in SSC, but to our knowledge no qualitative systematic review has been published. This literature review synthesizes findings from 29 original research papers from different countries and clinical settings.

## The review

### Aim

The aim with the qualitative systematic review was to describe parental experiences of providing SSC to their newborn infants.

### Design

The present qualitative systematic literature review was the first step of a meta-study guided by the methodology described by Paterson et al. (2001). The three first steps in the meta-study: a) formulating a research question, b) selecting and appraising primary research, and c) meta-data analysis are presented in this paper as a foundation for the meta-synthesis and interpretation resulting in a tentative theory (Paterson et al., 2001), which are presented in part 2. These two papers are the result of a Swedish-Brazilian research collaboration.

### Research question

The research question was: “How do parents of newborn infants experience performing SSC?” We decided to include original qualitative research about the experiences of all types of skin-to-skin intervention reported by mothers and fathers of newborn infants, irrespective of gestational age and hospital setting.

### Search methods

#### Systematic search

**Figure 1:**
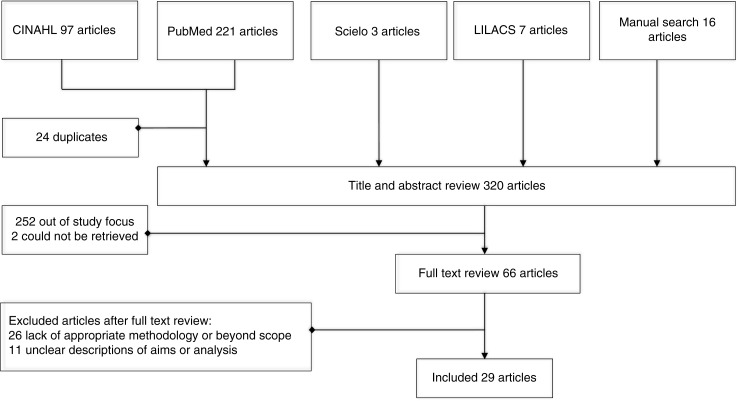
Flowchart showing the search and inclusion process.

In order to ensure an adequate scientific level, we limited the inclusion criteria to published original papers and doctoral dissertations. No language or publication year limitations were set. After screening subject headings (CINAHL), Mesh-terms (PubMed) and key words from some relevant manually identified articles, the search-terms Kangaroo, Kangaroo Care, Skin-to-skin, Parents, Parental attitudes, Parental behaviour, Infant care, Mother, Father and Parent–child relations were used in different combinations. The searches were performed in March 2009. Since the initial searches revealed extensive published research on this topic from Latin-America, further database searches were performed in SciElo and LILACS, two databases specialized in Latin-American research. To ensure actuality, the literature searches were repeated in June 2010 and in November 2013, using the same methodology (Figure 1).

#### Manual search

Manual searches were also performed in a bibliography compiled by Susan Ludington (personal communication), in reference lists and based on personal knowledge.

### Search outcome

Together the systematic searches resulted in 328 original papers of which 24 were duplicates. The manual search added a further 16 articles to be appraised for possible inclusion. Thus, the first set of articles for title and abstract review comprised 320 papers.

### Title and abstract review

As it was not possible to restrict the search to qualitative papers, the titles, and when available, the abstracts were scrutinized by each of the authors individually in order to identify papers that met the inclusion criteria. A large number of papers used quantitative methodology or focused on other aspects of newborn care, such as experiences of the neonatal intensive care unit (NICU) environment or breastfeeding, and were thus excluded from further analysis.

### Quality appraisal

Sixty-eight of the 320 papers were retained for the intended full text review. However, two of them could not be retrieved in full text, leaving 66 for further evaluation.

The authors read and appraised the full text of the 66 articles and compared their decisions pertaining to inclusion/exclusion. Each paper was scrutinized by 2–3 researchers in Sweden and Brazil (the articles in English and Portuguese, respectively) and, in one case, by a Japanese researcher (one article in Japanese).

All papers considered for inclusion were appraised by means of the Primary Research Appraisal Form presented by Paterson et al. (2001). This tool, developed from Burns’ (1989) statements about methodological congruence, was used to obtain structured information from and assess the methodological quality of the papers. The appraisal form is divided into sections describing the theoretical underpinnings, role and credentials of researchers, as well as research design, method, and major findings. Thereafter, the quality of the research and findings was appraised and a decision was made about whether or not to include the paper. To enable discussion and decision-making within the research group, the required information from all articles was translated into English.

As a result of the quality appraisal, 26 papers were excluded due to methodological reasons (being anecdotal or not containing a qualitative analysis of parental experiences) or for being beyond the scope of this review. Finally, an additional 11 papers were excluded because of flaws in the description of aims or analyses or because it was impossible to distinguish parental experiences from other findings (Table I). Most of the papers in this group were case studies including 1–2 cases and presenting the findings mainly in the form of quotations from parents. Finally 29 original papers were included, reflecting SSC in Brazil, Denmark, England, Japan, Norway, South Africa, Sweden, Uganda and he United States (Table II). Most of the participants in the included studies were mothers (*n*=401) while 94 fathers where included.

**Table I:** Excluded papers.

First author, year, country	Aim of the study	Theoretical orientation (T); Methodological orientation (M)	Population studied (P) Intervention (I)	Notes on findings	Methodological considerations and/or reasons for exclusion
Burkhammer, 2004, USA		T: Part of a larger study exploring possible reasons for breastfeeding difficulties. M: Case study.	P: 23-year-old mother, previous stillborn baby, GA 28, now SGA-baby GA 37 w, BW 2490 g. I: KMC was initiated after the immediate post-birth period.	This case study illustrates how KMC may help to facilitate emotional release and relaxation for the mother, enabling her to simultaneously grieve for her stillborn son and rejoice in the birth of her second son.	No clearly stated aim, theoretical orientation, or methodological description. No identification of limitations, categories, or framework.
Dombrowski 2000, USA		M: Case study, participant recruited from an RCT designed to investigate the effect of KMC on maternal and infant health based on (Moran, et al., 1999).	P: mother 17 y primipara, father 16 y. Twin boys GA 32 w, BW 1758 g and 1629 g. I: SSC 5–90 min, from 19 h to 5 d after birth, in NICU.	The mother expressed that it felt natural and that shared KC allowed the boys to feel relaxed and together. The father expereinced that the baby liked SSC, but as soon as the baby became uneasy, he believed it was hungry and gave it to the mother. However, he also stated that he knew what the baby liked.	No explicit aim. No analysis. No substantive findings, only observations by the authors and a few quotations. Some relationship between the researchers and family was apparent due to the use of “we.”
Dombrowski, 2001, USA	To describe how KMC may have helped the transition of the mother of a newborn preterm infant to motherhood despite challenging circumstances.	M: Case study, participant recruited from an RCT designed to investigate the effect of KMC on maternal and infant health based on (Moran et al., 1999).	P: mother 22 y, single, multiparous. Infant girl, GA 35 w. BW 2430 g. I: KMC 1–6 h, from 2 h after birth.	KMC helped with bonding, the mother felt closer to the baby than when holding her wrapped in blankets. Mother felt more secure and less anxious taking care of her baby and stated that KMC made it possible to forget everything and reduced stress.	Case study without a description of the data collection or analysis.
Erlandssson, 2008, Sweden	To describe the meaning of fathers’ lived experiences when taking care of their infant as the primary caregiver during the first hours after birth, when the infant was separated from the mother due to the latter's postoperative care.	M: Phenomenological design. Open interviews.	P: 15 fathers of healthy infants, GA 37–42 w. I: Fathers were the primary caregivers for 1–7 h after the birth, caring for the infant skin-to-skin, held wrapped or dressed or in a cot.	A movement towards father–child togetherness characterized by an immediate/ change as the father assumed increasing responsibility while getting to know his child.	The findings were not specifically related to SSC.
Messmer, 1995, USA	A pilot study addressing future problems of preterm infants.	T: The Continuity Hypothesis proposes that certain stimuli become familiar and salient to the foetus, and if presented repeatedly to the newborn, will have soothing and regulating effects (Ourth & Brown, 1961).	P: 13 mothers in an 8-bed NICU.	Anxieties and fears associated with the NICU diminished and confidence increased in their ability to care for the child. They became more prepared to assume responsibility for the infant's care after discharge.	Poor methodological quality. Recruitment, data collection, and analysis methods not reported. No qualitative analysis performed.
Meyer, 1999, USA	To document clinical experiences of KMC.	M: Case study.	P: Three mothers with breastfeeding difficulties. Full-term infants.	The clinical experiences suggest that it is worthwhile trying KMC to achieve successful breastfeeding.	Three personal stories with very little analysis. No theoretical orientation.
Moran, 1999, USA	To document KMC initiated 4.5 h post-birth with a healthy mother whose infant required initial stabilization in the NICU; to describe this family's experience and outline the nurses’ role in supporting this care.	M: Case study, part of a larger study with 6 m follow-up.	P: Married couple “in their mid-thirties,” 12 years of infertility. Infant boy, GA 32 w, BW 1953 g. I: KMC at the NICU, 4.5 h post birth.	KMC initiated early and administered for several hours for the first 5 days post birth seemed to be a satisfying experience for the parents.	Case study without description of data collection or analysis.
Parker, 2002, USA	To describe the experiences of adoptive parents who provided KMC to their critically ill preterm infant.	M: Case study.	P: Biological mother 15 y. No description of adoptive parents. Infant girl, GA 27 w, BW 917 g. I: KMC on High-Frequency Ventilation on day 3 and subsequently until discharge to home hospital on day 10.	Both parents felt an immediate and intense connection with their adoptive daughter and that they began to “know” her while providing KMC.	Case study without a description of data collection or analysis.
Price, 2005, United Kingdom	To improve awareness of breastfeeding and the importance of skin-to-skin contact between mother and baby at birth.	M: Action research with semi-structured audio-taped interviews.	P: Midwives and 8 mothers in a maternity unit. I: Skin-to-skin contact at birth.	The mothers’ relationships with their babies were enhanced. The SSC helped the mothers to endure painful experiences and felt natural. Appreciated being able to use own body to keep the baby warm.	The results from the interviews with the mothers were sparsely analysed and reported.
Silva, 2008, Brazil	To analyse the perceptions, experiences, and neonatal care behaviour of women in a KMC Program.	M: Observations, questionnaires, interviews.	P: 5 mother–baby dyads. BW 900–1350 g.	All mothers followed the prescribed activities and the period spent on the Program seems to have been used as a reflective moment in their lives.	Mixed methods. Difficulty to distinguish experiences from other findings.
Swinth, 2000, USA	To illustrate how one mother was assisted in sharing KMC with her newborn triplets.	M: Case study, the mother was cared for in a randomized control study in a KMC setting and expressed concern about being unable to spread her love between the three babies as well as her four older children.	P: Mother and her 3 baby boys, GA 35 w, BW 1336 g, 1736 g and 1882 g. I: 3 skin-to-skin sessions over 2 d from day 6 immediately after care-giving and bottle-feeding.	The shared kangaroo care relieved fears about being unable to love all three infants as well as four older children.	Case study without a description of the data collection and analysis.

Note: KMC = kangaroo mother care; SSC = skin-to-skin care; RCT = randomized controlled trial; SGA = small for gestational age; NICU = neonatal intensive care unit; BW = birth weight; GA = gestational age; y = year; w = week; m = month; d = day; h = hour; min = minute; g = gram.

**Table II:** Studies included in the analysis.

Author, year of publication, country of study	Aim of the study	Design, data collection, and analysis	Population studied (P) Exposure to SSC (E)	Major findings
Affonso et al., 1989, Sweden	To identify and compare themes based on the reactions of two groups of mothers, using a cognitive adaptation framework.	Exploratory, descriptive design. Individual semi-structured interviews on the unit during and after the infant's care. Deductive content analysis using the attachment framework.	P: Sub-group of 33 healthy mothers, mean age 26.5 y (16–37 y). Total study: 66 mothers providing or not providing SSC. 33 infants, mean GA 31.1 w (26–28 w). E: When taken out of the incubator, the infants were healthy, stable, and between 1 and 30 d.	According to the framework of the cognitive adaptation theory, the mothers searched for meaning and described a sense of mastery as well as self-enhancement
Affonso et al., 1993, USA	To explore the effects of KMC on mothers’ reactions	Individual semi-structured interviews. Deductive content analysis using the attachment framework.	P: 6 mothers (26–38 y). Infants GA 26–30 w, BW 765–1530 g in a NICU, Inclusion at 9–64 d. E: Minimum SSC 4 h/d, 6 d/w during 3 consecutive w.	SSC via the KMC method facilitates psychological healing and regaining the mothering role in an intensive care nursery.
Arivabene & Tyrrell, 2010, Brazil	To describe the mothers’ experiences of KMC, analyse them in the light of KMC principles and discuss the mothers’ contributions based on the meanings of their experiences of KMC and thus implications for nursing practice.	Focus groups	P: 13 mothers (18–40 y) with low socioeconomic status in a NICU. No data on infants provided.	Increased bonding between mother and baby, reduction of the infant's separation from the family, increased competence and confidence on the part of the parents even before discharge, improved relationship between the mother and the rest of the family, within the family, and with the team taking care of the baby.
Blomqvist & Nyqvist, 2010, Sweden	To investigate mothers’ experiences of KMC.	Retrospective survey. Content analysis.	P: 10 mothers answered the open-ended questions. E: The total eligible group of 23 infants GA 35.5 w, BW 2535 g, no data for the analysed sub-group.	The mothers’ experiences were predominantly positive. Negative comments concerned lack of information. Some mothers perceived the care during the night as exhausting.
Blomqvist et al., 2012, Sweden	To describe fathers’ experiences of providing their preterm infants with KMC.	Semi-structured interviews. Content analysis.	P: 7 first-time fathers, age 25–36 y. Preterm infants, GA 29–33 w, BV 1315–2500 g. E: KMC with father started day 1–5, between 211 and 478 min/d.	The fathers’ opportunity for being close to their infants facilitated attainment of their paternal role. They were active agents in their infant's care. The physical environment and conflicting staff statements influenced their experience.
Blomqvist et al., 2013, Sweden	To identify factors that parents of preterm infants perceived as supportive factors or barriers for their performance of KMC.	Retrospective survey. Content analysis.	P: 76 mothers, 74 fathers Preterm infants GA 31.8 (28.4–33.9) w, BW 1781 (740–2920) g. E: The NICUs faciltated and encouraged KMC up to 24 h/d but no information on the included infants exporsure to KMC is provided.	Four categories were identified regarding support and barriers for parents’ performance of KMC: parent-related factors, time, infant-related factors, and the NICU and home environment. The hospital staff and environment were described by the parents as both supportive and barriers for their application of KMC.
Braga et al., 2008, Brazil	To investigate perceptions and experiences of mothers of premature babies who breastfed exclusively from the 4th to the 6th month of life.	Individual open-ended interviews. Phenomenology.	P: 8 mothers aged 17–38 y. Infants GA<37 w, BW 1800–2500 g.	KMC is regarded as one of the factors that facilitate breastfeeding. One criterion is that the mother really wants to participate and has time, as the health care team must never impose the technique.
Byaruhanga et al., 2008, Uganda	To explore the perceptions of SSC among post-delivery mothers in order to identify factors that could influence the acceptability of this method.	Focus group discussions. Latent content analysis.	P: Sub-group of 30 mothers from another study, mean age 25 y, 18 multipara. Full-term infants. E: SSC after post-delivery bathing.	Acceptability of health practices influenced by knowledge and sensation. Pregnant women's choices dependent on social, cultural, and economic factors.
Caetano et al., 2005, Brazil	To understand family dynamics and transformation as a result of KMC.	Grounded theory. Interviews with open questions.	P: 18 mothers in a KMC unit, mean age 27.2 y. Infants mean GA 29.2 w, mean BW 1195 g. E: 6–45 d in a KMC unit. with KMC all the time (no cots in unit).	The lived experience consists of one central category: Weighing the risks and benefits between staying with the child in the kangaroo method or with the family, including three phenomena: 1) un-expected evolution and outcome in pregnancy, 2) coping with the prematurity of the child, 3) living with the decision and the experience together with the child.
Campos et al., 2008, Brazil	To explore the mothers’ perceptions of KMC.	Descriptive study with qualitative approach. Semi-structured interviews.	P: 13 mothers (19–39 y) in a KMC unit. No data on infants provided.	Strengthening of the bond between mother and newborn. Mothers recognize and appreciate the physical benefits for the infant and the opportunity to learn how to take care of a premature baby.
Dalbye et al., 2011, Norway, Sweden	To explore experiences of SSC in healthy mothers of healthy, full-term infants in the first days after birth.	Interviews. Phenomenology.	P: 3 primiparous and 7 multiparous women, age 24–37 y. Healthy full-term infants. E: First 2 h after delivery plus “as much as possible” first 24 h.	The SSC started a positive spiral. A mutual interaction developed which acted as a generator releasing energy to the mother. Happiness, peace and satisfaction were expressed by the newborns.
Duarte & de Sena, 2004, Brazil	To capture the mothers’ understanding of KMC and reveal the contradictions between the reality and their perceptions of the availability required to provide this care.	Descriptive exploratory qualitative study, guided by the dialectical method.	P: 15 mothers in a KMC unit. No data on infants provided.	KMC is an opportunity to recover the disbanded unit, favouring transition from a pregnant woman to a mother. KMC is a form of process that involves women's bodies and emotions, strengthens their bond with the infant, and is perceived as rewarding.
Eleutério et al., 2008, Brazil	To explore the perceptions of mothers who experienced KMC during hospitalization in the KMC infirmary.	Semi-structured interviews. Content analysis.	P: 9 mothers in a KMC unit. No data on infants provided.	Four themes: 1) knowledge, 2) care, 3) receptivity, 4) caress. The mothers considered the Kangaroo method an opportunity for learning how to care for their babies and that bonding is relevant and helps in the baby's recovery.
Finigan & Davies, 2004, England	To explore women's lived experiences of SSC with their baby immediately after birth. To investigate the experiences from the women's own perspective and establish whether or not this is a mother-friendly approach.	Audio diaries from birth up to 28th day post-partum. In-depth interviews. Thematic analysis.	P: 6 mothers (21–36 y), 5 multigravidas. E: SSC within 30 m of the birth and maintained for at least 1 h.	Five themes: 1) immediate feelings of bonding, 2) touch and stroking, 3) the gaze and getting to know the baby, 4) natural, instinctive behaviour, 5) not wanting to let go of the baby.
Furlan et al., 2003, Brazil	To analyse preterm babies’ parents’ perceptions of KMC, in order to introduce subsidies for the promotion of humanized assistance to support NICU clients.	Qualitative descriptive. Semi-structured interviews 60 days after discharge from the KMC unit. Thematic analysis.	P: 5 couples (5 mothers, 5 fathers) (18–33 y). Preterm infants, about 35 w and 1100–1500 g at start of KMC. E: KMC 8–12 h/d for 12–30 d.	Four thematic nuclei: 1) The flexibility of the maternal stay in the KMC ward, 2) Giving support to mother–child and family relationships, 3) Completing the growth and development of the premature infant, and 4) Developing skills to take care of the premature baby.
Heinemann et al., 2013, Sweden	To describe parents’ experiences of factors that influenced their stay with their extremely preterm infants in a NICU.	Qualitative descriptive. Semi-structured interviews at least 1 w after transfer to home or step-down unit. Content analysis.	P: 7 couples (7 mothers, 6 fathers). Preterm infants GA 23 w+5 d to 27 w+6 d, BW 492–1044 g. E: not specified but SSC for all infants was facilitated and encouraged at the NICU.	Two themes: 1) Coping with a new and unexpected situation, and 2) Becoming a parent.
Helth & Jarden, 2013, Denmark	To explore how fathers of premature infants experience and potentially benefit from using skin-to-skin method during the NICU stay.	Hermeneutic phenomenological. In-depth, semi-structured interviews.	P: 5 first-time fathers (28–37 y). Preterm infants GA<35 w. E: not specified but all fathers had SSC experience at the NICU.	Three themes: 1) The competent parenthood, 2) The paternal role and the division between the parents, 3) Balance between working life and time spent with the infant.
Johnson, 2007, USA	To describe mothers’ experience of kangaroo holding of premature infants in the neonatal intensive unit as a means of gaining insight into specific maternal benefits of this intervention.	Qualitative naturalistic inquiry. Open-ended interviews. Content analysis. Observations were also carried out, the results of which were combined with the interview data.	P: 18 primiparous mothers, mean age 26.3 y. Infants mean GA 28.8 w, mean BW 1410 g. E: Held infant for 60 min in the NICU on three occasions during the first 2 w of her/his life.	Three themes: 1) Maternal–infant benefits of kangaroo holding, 2) Need of support for holding, and 3) Satisfaction with interaction.
Lamy et al., 2011, Brazil	To reveal how women construct their maternal role when they have had a preterm and/or low birth weight infant in a NICU.	Semi-structured interviews.	P: 20 mothers from 4 hospitals, having their infant for 1–3 m in the NICU. No data on infants provided.	KMC helped the women to feel like and consider themselves mothers. They also expressed confidence in the baby and felt more certain in their maternal role.
Leonard & Mayers, 2008, South Africa	To explore the lived experiences of parents who provided their preterm infant with KMC.	A qualitative, explorative and contextual study in the phenomenological tradition. In-depth interviews.	P: 4 mothers, 2 fathers. Premature infants BW>1000 g,>1 w. E: Active provision of KMC in the neonatal nursery and KMC ward of a tertiary hospital.	Six themes: 1) unforeseen, unprepared and uncertain—the experience of birth, 2) anxiety and barriers, 3) an intimate connection, 4) adjustments, roles, and responsibilities, 5) measuring success, and 6) a network of encouragement and support.
Martins & Dos Santos, 2008, Brazil	To identify the mothers’ difficulties participating in KMC and observe the strategies they used to overcome the difficulties.	Qualitative descriptive design. A structured questionnaire with five open questions. Interviews. Thematic analysis.	P: 5 mothers (17–34 y) participating in KMC in an Intermediate Care Unit. Infants GA <37 w, BW <1250 g. E: KMC as soon as the infant was medically stable, continuously if possible.	The thematic analysis resulted in two categories: 1) learning how to be a kangaroo mother, 2) living as a kangaroo mother.
De Moura & Araújo, 2005, Brazil	To understand KMC users’ perceptions of the concept of motherhood and their motherhood experience.	Semi-structured interviews and observations. French Discourse Analysis based on Foucault and Guattari's notion of subjectivity.	P: 8 low-income mothers in hospital KMC unit. No data on infants provided. E: Holding baby in kangaroo position.	1) family and religion were characterized as central elements in attribution of meaning to motherhood, 2) the impact of premature birth, leading to disruption in the construction of the maternal role, 3) relationships with institutions and health professionals characterized by distrust and resistance, and 4) experience of KMC, which provided an opportunity to establish contact with the child andgain confidence in one's own mothering role.
Nakajima, 2002, Japan	To study the effect of kangaroo care on maternal attachment and healing.	Comparison analysis. Triangulation approach was used to compare the similarities and differences of the qualitative and quantitative results. Interviews analysed by means of comparison analysis.	P: 20 mothers who experienced KMC on more than three occasions. Premature infants BW<2500 g. E: KMC after an infant reached GA 32 w, up to 2 h/d.	Three themes: 1) feelings of guilt and uncertainly were alleviated, 2) mothers felt released from the constant feeling of hurt or pain, and 3) obtained a greater sense of “this is my child.”
Neu, 1999, USA	To explore parents’ perceptions of providing SSC to their preterm infant who was receiving assisted ventilation and elucidate factors that influenced the decision to continue or discontinue SSC.	Naturalistic inquiry. Interviews. Content analysis.	P: Sub-group of 8 mothers and 1 father from another project, mean age 25.9 y (21–37). 4 primiparous. Premature infants, mean BW 1064 g (SD 423), mean GA 27.2 (2.0) w. E: Two 60 m SSC sessions on consecutive days.	Three themes: 1) ambivalence of parents towards SSC, 2) need of a supportive environment, and 3) special quality of the parent–infant interaction.
Neu, 2004, USA	To describe factors that influenced mothers of healthy preterm infants to choose kangaroo holding rather than the standard blanket holding method.	Naturalistic inquiry. Interviews. Content analysis.	P: 24 primiparous mothers, median age 30 y (18–41). Healthy infants, median GA 32.5 w (31–34). E: The KMC regimen was standard in the NICU and also provided after discharge.	Three themes: 1) expression of emotional distress, 2) perception of a facilitative environment for holding, 3) perceived benefits of close contact with the infant.
Neves et al., 2010, Brazil	To identify mothers’ perceptions of KMC.	Semi-structured interviews.	P: 6 mothers in a KMC unit. Preterm, stable infants.	KMC made the mothers more familiar with their infant.
Roller, 2005, USA	To gain an understanding of mothers’ experiences of providing KMC for their preterm newborns.	Transcendental phenomenology. Semi-structured interviews, observations.	P: 10 mothers. Preterm infants, GA 32–26 w, BW 1500–3000 g. E: KMC within the first 24 h after birth at the neonatal unit.	Four main themes, which were reduced to one essential structure of knowing: mothers were prevented from knowing or getting to know their preterm newborn. Only one theme concerned the aim of our study; kangaroo care and also some parts of the theme Reassurance
Toma, 2003, Brazil	To increase understanding of the influence of hospital conditions and family organization on KMC practice.	Qualitative descriptive design. Interviews based on a guide.	P: 14 young mothers (10 first time) and 7 fathers. No data on infants provided.	The opportunity for effective parent participation from the beginning of life supports the creation and strengthening of the relationship and makes taking care of the child easier. However, the success of KMC does not only depend on the mothers’ will, but also on the support of family networks and of a comprehensive health care staff.
Toma et al., 2007, Brazil	To improve KMC by contributing to the knowledge of the different ways in which low-income families deal with a preterm baby.	Grounded theory. 2 interviews, on day of discharge and at home after 15–30 d. 3 open questions about pregnancy, childbirth as well as hospital and home postpartum periods.	P: 22 mothers, mean 26 y. (part of a larger sample containing an additional 19 mothers pre-intervention). Infants BW<2000 g who remained>1 w at the NICU.	The need to care for their other children appeared to be one of the main KMC constraints. The trend towards nuclear families hindered women's participation in the programme. Awareness of the limitations and possibilities of each family may contribute to improved implementation.

Note: KMC=kangaroo mother care; SSC=skin-to-skin care; NICU=neonatal intensive care unit; BW=birth weight; GA=gestational age; y=year; w=week; m=month; d=day; h=hour; min=minute; g=gram.

### Data extraction

Data related to parental experiences were retrieved from quotations in the original papers, as well as from the findings sections in these papers. All relevant data were extracted and copied into a “Citations and findings extract template,” which in the further process was regarded as the primary data for analysis.

### Meta-data analysis

The meta-data analysis synthesizes data from the text of the included literature. The same data analysis techniques that can be used in primary research are also applicable to meta-data-analysis (Paterson et al., 2001). Thus, qualitative content analysis (Graneheim & Lundman, 2004) was chosen as a strategy for the meta-data analysis. The analysis was performed by means of the nVivo 8.0 (QSR International, Doncaster, Victoria, Australia) software. All findings from the “Citations and findings extract template” were imported into the software database and were coded and categorized by three researchers. Based on similarity of content, the codes were collapsed into descriptive manifest categories. Thereafter, sub-themes revealing an interpretative level of content were searched for. Finally, two themes were identified, based on the content of the 5 sub-themes and 19 categories (Table III). During the entire process, discussions were held between the research groups in Sweden and Brazil.

**Table III:** Overview of themes, sub-themes, and categories.

Themes	A restorative experience			An energy-draining experience	

Sub-themes	Feeling good	Doing good	Becoming us	Feeling exposed	Hurting others
Categories	A heart-warming experience Relieving emotional suffering A rewarding experience A natural instinct A learning experience Finding a role Improved self-esteem Feeling of control A supportive environment	A way of knowing and understanding Important for the infant	A bonding experience Intimate togetherness	Environment as an obstacle A physical and emotional burden Incongruence between wishes and demands Uncertainty about the purpose of and own skill in providing SSC	Fear of hurting Feeling insufficient towards the family

## Results

### A restorative experience

The common latent content of the categories in this theme are the positive and restorative components of the parental experience of providing SSC. Feeling good is the dominant part found in the literature, but the theme also includes experiences of doing good for the infant and a sense of becoming unified as a family.

### Feeling good

#### A heart-warming experience

Mothers described an instant and overwhelming love on the occasion of their first skin-to-skin contact with their infant (Finigan & Davies, 2004; De Moura & Araújo, 2005). They reported that they melted the first time the infant looked at them (Finigan & Davies, 2004) and that skin-to-skin holding was a wonderful experience (Neves, Ravelli, & Lemos, 2010; Roller, 2005). Furthermore, mothers and fathers expressed a very special and unique sense of joy and happiness (Affonso Bosque, Wahlberg, & Brady, 1993; Byaruhanga, Bergstrom, Tibemanya, Nakitto, & Okong, 2008; Campos, Carvalho, Rolim, & Alencar, 2008; Dalbye, Calais, & Berg, 2011; Finigan & Davies, 2004; Furlan, Scochi, & Furtado, 2003; Helth & Jarden, 2013; Johnson, 2007) and warmth (Eleutério, Rolim, Campos, Frota, & Oliveira, 2008; Furlan et al., 2003). Some considered such strong emotions being a gift from God (Furlan et al., 2003).

One woman described experiencing a strange feeling when her infant was placed on her chest immediately after delivery. The mother felt sweaty and wet, yet appreciated the experience (Finigan & Davies, 2004). Furthermore, mothers described a sense of calm and peace (Dalbye et al., 2011) and feeling relaxed when providing SSC to their infant (Affonso, Wahlberg, & Persson, 1989; Heinemann, Hellstrom-Westas, & Hedberg Nyqvist, 2013; Neu, 2004; Roller, 2005). In a neonatal setting as well as in delivery care fascination was described by the infant's movements and competence in exploring the world by looking around (Affonso et al., 1989; Finigan & Davies, 2004). Parents were found to be delighted at being able to watch their premature infant's development, which should have been taking place in utero (Leonard & Mayers, 2008) and to sense the little heart beating (Neves et al., 2010). The immediate sense of love and compassion for the infant was accompanied by reduced guilt, anguish (Campos et al., 2008; De Moura & Araújo, 2005), fear, and rejection (De Moura & Araújo, 2005).

#### Relieving emotional suffering

SSC enabled the mothers to acknowledge their innermost feelings, elaborate on the experience of giving birth to a premature infant and find meaning in the situation (Affonso et al., 1989). To see the infant's strength, eased the mother's pain caused by the infant's condition (Nakajima, 2002) as well as her feeling of guilt (De Moura & Araújo, 2005; Nakajima, 2002) and fear (De Moura & Araújo, 2005). SSC made parents hope that everything would be all right (Braga, Machado, & Bosi, 2008; Caetano, Scochi, & Angelo, 2005; Furlan et al., 2003) and that the infant would survive (Campos et al., 2008).

#### A rewarding experience

Mothers described SSC as a reward (Duarte & de Sena, 2004; Finigan & Davies, 2004) that they would not like to forego (Finigan & Davies, 2004). SSC was also characterized as meeting mothers’ need for affection (De Moura & Araújo, 2005) and providing them with well-being (Campos et al., 2008) and energy (Dalbye et al., 2011).

#### A natural instinct

Parents revealed that SSC was a natural instinct (Affonso et al., 1989; Byaruhanga et al., 2008; Caetano et al., 2005; Finigan & Davies, 2004; Neu, 1999). In one study, being natural was related to a desire to protect and do everything possible for the infant (Caetano et al., 2005), also expressed as the right thing to do from the infant's perspective (Finigan & Davies, 2004).

#### A learning experience

Mothers who provided SSC described that it taught them how to be a mother (Johnson, 2007) and made them accustomed to handling their infant (Affonso et al., 1993; Campos et al., 2008; Eleutério et al., 2008; Furlan et al., 2003; Lamy et al., 2011; Martins & Dos Santos, 2008; Toma, 2003; Toma, Venancio, & Andretto, 2007) and to breastfeed (Braga et al., 2008; Toma et al., 2007). Experience in taking care of the infant prepared the mothers to assume full responsibility after discharge from the hospital (Affonso et al., 1989, 1993; Furlan et al., 2003; Lamy et al., 2011; Leonard & Mayers, 2008). Leonard and Mayers’ (2008) study described that fathers wanted to be instructed in kangaroo care by the mothers, not by professionals. One father said that since he never played with dolls as a child, this was a new and pleasant learning experience for him (Helth & Jarden, 2013).

#### Finding a role

Mothers felt the need to take responsibility for something when their infant was hospitalized due to prematurity and SSC fulfilled this need (Affonso et al., 1993; Johnson, 2007; Roller, 2005). Johnson (2007), Leonard and Mayers (2008), and Campos et al. (2008) found that SSC allowed parents to experience being a part of the infant's care process. Other authors found that SSC facilitated mothers to assume a mother role (Affonso et al., 1989, 1993; Furlan et al., 2003; Leonard & Mayers, 2008; Neu, 1999) and increased their confidence (Affonso et al., 1993; Furlan et al., 2003). Providing the infant with milk meant being part of the beneficial process of the child's growth and development (Leonard & Mayers, 2008). De Moura and Araújo (2005) argued that SSC had an impact on mothers’ social identity, as motherhood in Brazil is highly valued and essential in the construction of a woman's social identity. SSC also helped the women's partners in starting to assume the father role (Affonso et al., 1993; Blomqvist, Rubertsson, Kylberg, Joreskog, & Nyqvist, 2012; Finigan & Davies, 2004; Helth & Jarden, 2013; Leonard & Mayers, 2008; De Moura & Araújo, 2005; Neu, 1999).

Mothers described that providing SSC made them feel needed by the nurses on the ward (Johnson, 2007), although they also valued the care given to the infant by the nurses (Campos et al., 2008).

#### Improved self-esteem

From a general perspective it was found that providing SSC improved parents’ self-esteem (Blomqvist & Nyqvist, 2010; Finigan & Davies, 2004; Helth & Jarden, 2013; De Moura & Araújo, 2005). This was seen as a process (Affonso et al., 1989, 1993; Campos et al., 2008; Leonard & Mayers, 2008; Martins & Dos Santos, 2008; Nakajima, 2002; Neu, 1999; Roller, 2005). The areas in which self-esteem was improved were breastfeeding capacity (Affonso et al., 1989) and taking care of the infant (Affonso et al., 1989, 1993; Campos et al., 2008; Furlan et al., 2003; Helth & Jarden, 2013; Lamy et al., 2011; Nakajima, 2002). A sense of security that they were as good as the monitors for controlling the child's vital signs and meeting her/his needs was also described. As their self-esteem improved they no longer considered technical equipment frightening (Affonso et al., 1993).

#### Feeling of control

SSC gave mothers and fathers a sense of control (Affonso et al., 1993; Helth & Jarden, 2013; Roller, 2005). Mothers were experiencing immediate access to the infant (Byaruhanga et al., 2008; Campos et al., 2008 #4456; Duarte & de Sena, 2004 #4537; De Moura & Araújo, 2005 #3586; Roller, 2005 #4625; Toma et al., 2007 #3596) which meant first-hand information about her/his condition (Campos et al., 2008; Duarte & de Sena, 2004; De Moura & Araújo, 2005; Roller, 2005; Toma et al., 2007), which in turn made them more relaxed (Campos et al., 2008; Furlan et al., 2003). They were able to follow the infant's development (Furlan et al., 2003; Neves et al., 2010). The SSC reduced the mothers’ fear related to the infant and the NICU-environment. As a consequence, mothers visited the infant more frequently and experienced the technical equipment as a valuable tool for monitoring the infant's health in her own absence (Affonso et al., 1989). SSC reassured mothers that the infant's ability to breastfeed was improving (Affonso et al., 1989; Campos et al., 2008; Neves et al., 2010; Toma, 2003).

#### A supportive environment

The restorative aspect of SSC embraced experiences of an enabling environment leading to a more positive experience of SSC as well as an increased well-being in the parents; they felt good about the experience. This was expressed as a positive and encouraging attitude from the professionals that made it easier for the parents to provide SSC (Campos et al., 2008; Leonard & Mayers, 2008; Neu, 1999; Neves et al., 2010). In addition, practical assistance when providing SSC was experienced as valuable (Blomqvist & Nyqvist, 2010; Campos et al., 2008; Lamy et al., 2011; Neu, 2004). Mothers described the nurses as care-providers for both themselves and the infant (Campos et al., 2008; Neu, 2004). When providing SSC, mothers experienced themselves as VIPs on the ward (Blomqvist, Frolund, Rubertsson, & Nyqvist, 2013; Campos et al., 2008). They appreciated when nurses and technical staff were kind, quiet, and understanding (Campos et al., 2008) and reported that they relied on the staff (Lamy et al., 2011).

Accessibility was described as essential for successful SSC (Neves et al., 2010) and, furthermore, a comfortable environment was also important (Affonso et al., 1989; Blomqvist et al., 2013; Furlan et al., 2003; Heinemann et al., 2013). Good food, clean surroundings, and the possibility to watch TV while staying with their infant was also highlighted (Furlan et al., 2003). In one study, support from other mothers who had been in the same situation was stated to be of great value (Lamy et al., 2011). From a Swedish study even the government was seen as supportive, providing social benefits that allowed the parents to take a leave from work to be with their newborn at the NICU (Blomqvist et al., 2013).

Mainly in Brazilian studies (Arivabene & Tyrrell, 2010; Caetano et al., 2005; Furlan et al., 2003; Lamy et al., 2011; Martins & Dos Santos, 2008; Toma, 2003; Toma et al., 2007), but also in a study from Sweden (Blomqvist et al., 2012), the importance of family support was highlighted. Mothers described how visits from the father made SSC easier (Caetano et al., 2005; Martins & Dos Santos, 2008) and that fathers helped in the home while they were at the hospital providing SSC (Toma et al., 2007). Furthermore, it was revealed that if the father could manage to be present at the hospital, they could share SSC, which made the mother feel at ease (Toma, 2003). Mothers also valued support from other relatives (Arivabene & Tyrrell, 2010; Lamy et al., 2011; Martins & Dos Santos, 2008; Toma, 2003; Toma et al., 2007). One study reported that the local church provided support by organizing volunteers to help older children with their homework and assisting in the home, which made SSC in the hospital easier for the mother. Support from neighbours was also mentioned as positive (Arivabene & Tyrrell, 2010), while two studies reported the importance of religious belief (Arivabene & Tyrrell, 2010; Lamy et al., 2011).

### Doing good

#### A way of knowing and understanding

SSC was one means of becoming familiar with the infant by noticing and interpreting her/his signs (Affonso et al., 1989, 1993; Blomqvist et al., 2013; Eleutério et al., 2008; Johnson, 2007; Neu, 2004; Neves et al., 2010). Mothers experienced that they became even better at this than the nurses and technical equipment. More specifically, they reported that they became accustomed to the infant's breathing (Affonso et al., 1989; Leonard & Mayers, 2008; Neves et al., 2010) and knew what to do if it diverged from the normal pattern (Affonso et al., 1989). Furthermore, they learned to recognize signs of hunger and became more aware of the infant's body temperature and sleep pattern (Neves et al., 2010). SSC reduced the parents’ sense of their infants being fragile and thus their anxiety about caring for her/him (Affonso et al., 1993; Leonard & Mayers, 2008; Toma, 2003). As mothers and fathers got to know the infant by means of SSC, they also learned to recognize her/his competence (Blomqvist et al., 2012; Furlan et al., 2003; Johnson, 2007; Lamy et al., 2011) and sensed her/his strength of life (Nakajima, 2002).

#### Important for the infant

Parents described SSC as important for the infant's recovery (Johnson, 2007; De Moura & Araújo, 2005; Nakajima, 2002). Providing SSC was one way to help the infant to develop (Arivabene & Tyrrell, 2010) and survive (Arivabene & Tyrrell, 2010; Caetano et al., 2005) as well as a method of establishing breastfeeding (Braga et al., 2008; Byaruhanga et al., 2008; Campos et al., 2008; Eleutério et al., 2008; Leonard & Mayers, 2008; Neves et al., 2010; Toma et al., 2007). Providing SSC was seen as one way for the infant to gain weight (Arivabene & Tyrrell, 2010; Braga et al., 2008; Caetano et al., 2005; Campos et al., 2008; Furlan et al., 2003; Leonard & Mayers, 2008; Martins & Dos Santos, 2008; Neves et al., 2010; Toma, 2003), thereby offering possibilities for a quicker discharge from hospital (Arivabene & Tyrrell, 2010; Braga et al., 2008; Campos et al., 2008; Furlan et al., 2003; Neves et al., 2010).

Furthermore, the warmth established by SSC was considered important for recovery (Blomqvist et al., 2013; Duarte & de Sena, 2004; Eleutério et al., 2008; Leonard & Mayers, 2008; Neves et al., 2010; Toma et al., 2007), as was the skin contact in itself (Eleutério et al., 2008). The infant's oxygen saturation was reported to be increased (Neu, 1999) and the infant was experienced as being more chubby, more alert, and having a healthier skin colour (Toma et al., 2007). It also made the infant more comfortable (Affonso et al., 1993; Blomqvist et al., 2012; Dalbye et al., 2011; Leonard & Mayers, 2008; Toma, 2003) and calmer (Caetano et al., 2005; Eleutério et al., 2008; Lamy et al., 2011; Neves et al., 2010; Roller, 2005). One mother in the study by Neu (2004) described how the baby settled and constantly smiled until he fell asleep when in the skin-to-skin position. Mothers and fathers also viewed SSC as a way of protecting the infant (Caetano et al., 2005; Campos et al., 2008; Helth & Jarden, 2013; Lamy et al., 2011), particularly from infections (Braga et al., 2008) and stated that it made the infant feel secure (Finigan & Davies, 2004; Lamy et al., 2011; Neu, 1999; Toma et al., 2007).

Mothers emphasized the importance for the infant of knowing that she was there for her/him (Lamy et al., 2011; Neu, 1999). SSC allowed the infant to smell the mother and to be held in a different way than by the nurses. SSC was regarded as transferring affection (Arivabene & Tyrrell, 2010; Campos et al., 2008; Eleutério et al., 2008), strength, courage, and hope to the infant (Leonard & Mayers, 2008). Furthermore, in one study SSC was described as important for infants other than one's own baby, as it freed incubators, which other infants might require (Braga et al., 2008).

Although good for the infant, providing SSC was experienced as difficult when the infant underwent blood tests (Arivabene & Tyrrell, 2010). Nevertheless, mothers accepted the need for some sacrifices, as they recognized the benefits of SSC for their infant (Braga et al., 2008; Campos et al., 2008; Duarte & de Sena, 2004; De Moura & Araújo, 2005). Apart from the family at home, their own career was sacrificed by their dedication to SSC (Arivabene & Tyrrell, 2010). Fathers expressed that it was natural to support the mothers in providing SSC by accepting that they paid less attention to their normal duties at home, a sacrifice considered natural because of the infant (Furlan et al., 2003).

### Becoming us

#### A bonding experience

A sense of mutuality in getting to know each other (mother—infant) was described as emanating from SSC (Affonso et al., 1989; Dalbye et al., 2011; Eleutério et al., 2008; Finigan & Davies, 2004; Furlan et al., 2003; Johnson, 2007).

As the parents became familiar with their baby by means of SSC, they reported entering into a relationship with him/her (Affonso et al., 1989, 1993; Dalbye et al., 2011; Eleutério et al., 2008; Finigan & Davies, 2004; Furlan et al., 2003; Leonard & Mayers, 2008; De Moura & Araújo, 2005; Neu, 1999; Roller, 2005). They felt closer to their infant (Arivabene & Tyrrell, 2010; Byaruhanga et al., 2008; Campos et al., 2008; Duarte & de Sena, 2004; Neves et al., 2010; Roller, 2005; Toma et al., 2007) and created a unique connection (Leonard & Mayers, 2008; De Moura & Araújo, 2005; Nakajima, 2002; Neu, 1999, 2004; Toma, 2003). This special bond was described as making parents love their infant (Byaruhanga et al., 2008; Finigan & Davies, 2004), despite the pain the infant caused the mother during labour (Byaruhanga et al., 2008). It also strengthened fathers’ overall experience of labour (Finigan & Davies, 2004).

#### Intimate togetherness

It was reported that the SSC strengthened the sense of being a family (Braga et al., 2008; Dalbye et al., 2011; Finigan & Davies, 2004; Furlan et al., 2003; Heinemann et al., 2013; Johnson, 2007). Providing SSC was described as involving both parents, as they took it in turns (Toma, 2003). It was also seen as a family issue where the mother taught the father (Leonard & Mayers, 2008) or the parents supported each other in providing SSC and were both available to the infant (Caetano et al., 2005; Dalbye et al., 2011). Mothers described that during SSC their entire focus was on the infant (Finigan & Davies, 2004; Johnson, 2007). SSC was reported to provide a better bonding experience than breastfeeding (Roller, 2005) or any other kind of holding (Johnson, 2007).

Finally, the infant became familiar with the mother thanks to the SSC (Campos et al., 2008; Lamy et al., 2011). The infant's attachment was described in terms of being able to smell (Affonso et al., 1989; Furlan et al., 2003; Lamy et al., 2011; Leonard & Mayers, 2008; Neves et al., 2010; Roller, 2005) and touch the mother, thereby knowing that she/he was with her/his mother (Affonso et al., 1989; Furlan et al., 2003; Lamy et al., 2011; Leonard & Mayers, 2008; Roller, 2005). Once this contact had been established, mothers stated that the infant wanted to continue SSC with the parents, instead of lying alone in a cot or pram (Arivabene & Tyrrell, 2010).

### An energy-draining experience

The SSC was not only described in terms of being a restorative experience; it was also considered as energy-draining. The parents at times felt exposed when providing SSC and they were afraid of hurting others, primarily the infant, but also older children and family.

### Feeling exposed

#### Environment as an obstacle

There were many reasons why the environment was experienced as an obstacle. First, lack of autonomy was described; Mothers of full-term babies who provided SSC on the delivery ward experienced that their autonomy were not respected when they did not want to practise SSC but the midwife wanted them to do so (Byaruhanga et al., 2008).

Mothers expressed that it was difficult to practise SSC at the hospital, but easier at home where there were no spectators (Leonard & Mayers, 2008). Some reported unease at having to expose their body on the ward (De Moura & Araújo, 2005; Neu, 1999). Fathers described feeling critically assessed by staff when providing SSC, something that made them feel incompetent (Blomqvist et al., 2012; Helth & Jarden, 2013; Leonard & Mayers, 2008). The absence of a private bathroom was also negative in terms of privacy (Eleutério et al., 2008).

Inadequate privacy and lack of control (Blomqvist et al., 2013; Heinemann et al., 2013; Neu, 1999) were described as factors that made parents discontinue SSC. Other negative aspects were a physical environment with noise, hectic activity (Blomqvist et al., 2013) and sterility, as well as lack of support (Blomqvist & Nyqvist, 2010; Heinemann et al., 2013; Neu, 2004). Lack of information about the practical application of SSC was described as an obstacle (Blomqvist & Nyqvist, 2010; Dalbye et al., 2011), while technical equipment distracted or frightened parents and made them (Blomqvist & Nyqvist, 2010; Blomqvist et al., 2013; Neu, 2004), and thereby their infant, tense (Neu, 2004). When mothers felt that it was cold on the ward, they found it harder to take a break from SSC (Neves et al., 2010), or to initiate SSC, not wanting to undress the infant (Dalbye et al., 2011).

There were negative statements regarding family rooms, such as that they were too small and the beds uncomfortable (Blomqvist & Nyqvist, 2010; Blomqvist et al., 2012). Mothers requested furniture that would facilitate SSC, such as comfortable beds and armchairs, as they found it tiring having to remain in a sitting position all day (Braga et al., 2008; Furlan et al., 2003). Others wished for some form of activities to distract them, as they missed their home (Eleutério et al., 2008).

In two of the Latin-American studies, parents highlighted that travels to and from the hospital to provide SSC was a financial burden (Furlan et al., 2003; Toma et al., 2007) and in one study it was reported that the distance between the hospital and the family home made it more difficult to provide SSC on a daily basis (Neves et al., 2010). Negative remarks from relatives sometimes were seen as an obstacle to SSC (Blomqvist et al., 2012; Dalbye et al., 2011).

#### A physical and emotional burden

Parents described suffering from backache (Duarte & de Sena, 2004; Leonard & Mayers, 2008; Toma et al., 2007), being bored or tired, and experiencing anxiety when providing SSC in a KMC ward. Sleeping with the baby was experienced as difficult, because of the mother being woken up during the night (Leonard & Mayers, 2008; Neves et al., 2010), the responsibility involved (Blomqvist & Nyqvist, 2010), and having to sleep in a sitting position (Braga et al., 2008).

Providing SSC for many hours each day was also described as hindering other activities (Blomqvist et al., 2013; Duarte & de Sena, 2004; Leonard & Mayers, 2008; De Moura & Araújo, 2005; Neves et al., 2010). It was so difficult to visit the toilet or find time to eat that at times these needs were put aside, instead of taking a break from SSC (Neves et al., 2010). Providing SSC on a 24-h basis also had an impact on the mothers’ relationships outside the hospital and they experienced loneliness (Leonard & Mayers, 2008). Some mothers expressed frustration, feeling imprisoned at the hospital (Toma, 2003; Toma et al., 2007), and thereby in need of support from relatives to take care of their other children (Lamy et al., 2011; Toma, 2003; Toma et al., 2007). SSC was also experienced as tiring (Affonso et al., 1993; Blomqvist et al., 2012; Braga et al., 2008; Furlan et al., 2003; Toma et al., 2007) and stressful (Martins & Dos Santos, 2008).

The first attempt with SSC made some mothers tense and uncomfortable (Roller, 2005). Some felt more at ease and less stressed when holding the infant wrapped in a blanket, (Neu, 1999) and yet others experienced that the staff treated them as if they had no idea what they were doing (Neu, 2004). Another reason for feeling uncomfortable with SSC was that the mothers were afraid of bonding with their fragile infant (Nakajima, 2002). Although SSC facilitated breastfeeding in many cases, breastfeeding difficulties have been reported (Toma et al., 2007).

However, although experienced as uncomfortable at times, mothers considered SSC necessary (Braga et al., 2008; Duarte & de Sena, 2004; Neves et al., 2010) and some described it in terms of sacrificing their own needs for the infant's wellbeing and safety (Duarte & de Sena, 2004; Leonard & Mayers, 2008; Neves et al., 2010).

#### Incongruence between wishes and demands

Parents described that their own wishes were not always in line with the demands placed on them, by themselves or by others. They may have wanted to do more SSC than allowed to (Caetano et al., 2005; Finigan & Davies, 2004; Heinemann et al., 2013; Helth & Jarden, 2013; Johnson, 2007; Neu, 1999) or take a break, but felt obliged to continue (Blomqvist & Nyqvist, 2010; Blomqvist et al., 2013; Furlan et al., 2003; Martins & Dos Santos, 2008; Toma et al., 2007). In some cases, staff explicitly stressed the needs of and benefits to the infants (Arivabene & Tyrrell, 2010; Blomqvist & Nyqvist, 2010; Neu, 2004 #3589; Toma, 2003). In two studies, it was stated that mothers were allowed to take a break if feeling emotionally exhausted (Affonso et al., 1993; Blomqvist & Nyqvist, 2010). This was appreciated and in one of these studies it was reported that, on returning, the mothers were more motivated to continue SSC (Affonso et al., 1993). Fathers stated that they preferred providing SSC at home after the infant had been discharged, instead of at the hospital, where they felt exposed to spectators (Leonard & Mayers, 2008). On the contrary, fathers who were prevented from providing SSC for organizational reasons were frustrated and helpless, as they could not interact with their infant. One father described observing another father providing SSC until he was told to stop by staff members, an approach he considered too harsh (Leonard & Mayers, 2008).

In studies from a maternity ward setting, both parents expressed that they wanted to provide SSC, not just the mother (Finigan & Davies, 2004). However, some mothers did not like having their naked infant skin-to-skin after delivery, but wanted her/him washed and dressed beforehand (Byaruhanga et al., 2008).

#### Uncertainty about the purpose of and own skill in providing SSC

At times, parents did not understand the purpose of SSC (Byaruhanga et al., 2008; Leonard & Mayers, 2008; Toma, 2003), and in the delivery setting it was in some cases believed to be a trick to distract mothers who had to be sutured after a vaginal delivery (Byaruhanga et al., 2008). The parents were also uncertain about their own skill in providing SSC, which was especially obvious at the start of SSC (Leonard & Mayers, 2008; Martins & Dos Santos, 2008; Neu, 1999), but also in general (Eleutério et al., 2008; Martins & Dos Santos, 2008). They therefore expressed a need for guidance about how to hold the infant (Affonso et al., 1989; Johnson, 2007; Martins & Dos Santos, 2008). Toma (2003) highlighted the importance of supportive staff for successful SSC. Fathers thought that the mothers were superior in providing SSC and that the infant would not feel as comfortable with them. This assumption was based on a belief that the infant preferred the mother's smell (milk) and that the male body was not as suitable for SSC in a traditional position. However, some stated that they found alternative ways of providing SSC (Leonard & Mayers, 2008).

### Hurting others

#### Fear of hurting

Many studies found that parents were afraid of hurting their infant when providing SSC (Affonso et al., 1993; Blomqvist et al., 2013; Braga et al., 2008; Byaruhanga et al., 2008; Dalbye et al., 2011; Eleutério et al., 2008; Helth & Jarden, 2013; Johnson, 2007; Leonard & Mayers, 2008; Martins & Dos Santos, 2008; Nakajima, 2002; Neu, 1999, 2004; Toma, 2003; Toma et al., 2007). Mothers of full-term infants in Uganda were afraid of infecting their infants by providing SSC immediately after birth (Byaruhanga et al., 2008), while parents of premature infants feared hurting the baby as she/he was so tiny and worried about dislodging the infant's tubes (Neu, 1999). Parents were also afraid of dislocating their infant's legs (Neu, 2004). Furthermore, a fear of suffocating the infant was described when she/he was attached to one's own body (Braga et al., 2008).

When an early SSC session ended with the infant being negatively affected, it created fear of providing more SSC (Neu, 1999). Worry about hurting the infant decreased over time as the parents continued to provide SSC (Johnson, 2007; Martins & Dos Santos, 2008), although some parents discontinued SSC due to feeling very anxious (Neu, 1999). Parents not only feared physically hurting the infant but also disturbing her/him emotionally (Leonard & Mayers, 2008). Mothers described that at times their baby was uncomfortable with SSC (Neu, 1999; Toma et al., 2007).

#### Feeling insufficient towards the family

Mothers and fathers described feeling that they were neglecting the infant when staying with their family instead of providing SSC in the hospital (Caetano et al., 2005). They also reported feeling inadequate in meeting the needs of their other children (Arivabene & Tyrrell, 2010; Blomqvist & Nyqvist, 2010; Blomqvist et al., 2012 Blomqvist et al., 2013; Caetano et al., 2005; Campos et al., 2008; Dalbye et al., 2011; Duarte & de Sena, 2004; Lamy et al., 2011; Leonard & Mayers, 2008; Toma et al., 2007), husband (Arivabene & Tyrrell, 2010; Caetano et al., 2005; Campos et al., 2008; Duarte & de Sena, 2004; Johnson, 2007; Leonard & Mayers, 2008; Martins & Dos Santos, 2008), or parents (Duarte & de Sena, 2004). When reflecting on the tension between the infant and her/his older siblings, the mothers compared the children's need of them; the infant did not know them (Toma, 2003) and was taken care of by professionals in the hospital (Caetano et al., 2005), while older children knew and missed them (Caetano et al., 2005; Toma, 2003). However, mothers felt that the infant needed them more than the older children, who were healthy and thereby had less need (Caetano et al., 2005; Campos et al., 2008; Lamy et al., 2011). One mother described a feeling of tension when having to decide which twin needed SSC the most as there was only room for one in the “bag” used when providing SSC. Similar to the reasoning about older siblings, the mother opted to care for the smallest infant, as he/she needed more attention (Neves et al., 2010).

Parents reported that a consequence of the mother's SSC was that it changed family routines (Arivabene & Tyrrell, 2010; Caetano et al., 2005; Furlan et al., 2003; Martins & Dos Santos, 2008; Toma et al., 2007). At times the fathers had to assume responsibility for older children (Blomqvist et al., 2012; Caetano et al., 2005) or household tasks that they usually did not do (Toma et al., 2007). Fathers also had to engage help at home in order to stay with the mother and support her when providing SSC (Caetano et al., 2005) or expressed an accepting attitude that the mother spent less time on household chores (Furlan et al., 2003). However, being away from the family, knowing that her husband and children had to struggle at home, in combination with worries about the infant, influenced SSC (Arivabene & Tyrrell, 2010).

## Discussion

The findings offer a broad spectrum of nuances of parental experiences of providing SSC to their newborn infant, which have not been summarized before.

It is possible to trace some similarities between our findings and the results from studies conducted with parametric outcome scales. Below we will try to illuminate some issues where the qualitative findings can be seen as converging and complementing some of the previous results based on quantitative methodology (Heale & Forbes, 2013). In the majority of the included studies in this systematic review, experiences of being important for the infant were found. Such experiences were, for example, increased temperature control and increased growth, similar to results found by Charpak et al. (2005) and shown in the meta-analysis by Conde-Agudelo et al. (2011). Parents also experienced that by providing SSC they could influence the duration of hospital stay, converging the findings from the meta-analysis (Conde-Agudelo et al., 2011). However, the findings in this present systematic review complement the findings of a shorter duration in hospital by offering an understanding of the mothers’ expereince of this phenomena; By taking care of the infant, the mothers experienced themselves as prepared to assume full responsibility after discharge from the hospital. Together, the findings of shorter duration of hospital stay and the readiness to assume a full responsibility at discharge offers a more complete picture and understanding of the mothers’ situation, than just one of these perspectives.

The fact that there are similarities in the parental experiences of their own importance for the infants improvement, and in results describing the effect of SSC is interesting. Is it so, that the parents are informed by the professionals about previos benefitial research results, and adapt to them? This seems reasonable, as it was described that in some cases, staff explicitly stressed the needs of and benefits to the infants in order to motivate parents to perform SSC.

In infants with a low birth weight, meta-analyses demonstrated improved rates of breastfeeding and exclusive breastfeeding (Conde-Agudelo et al., 2011). Furthermore, mothers in the SSC-groups breastfed exclusively to a greater extent at hospital discharge (Cattaneo et al., 1998; Marín Gabriel et al., 2010). SSC was also in this systematic review seen as a method to facilitating breastfeeding. To succeed with breastfeeding was experienced as leading to an improved self-esteem, and by providing the infant with milk it meant being part of the beneficial process of the child's growth and development. However, although the SSC was described as facilitating breastfeeding, breastfeeding difficulties were reported in one study.

Another pattern in the results was that the parents expressed that SSC was related to bonding and attachment between the parent and the infant. Also, in studies with quantitative methodology SSC was found to have positive effects on mother-infant attachment/bonding (Charpak et al., 2005; Conde-Agudelo et al., 2011). A feeling of becoming a family was identified in the findings. This can be seen as related to the results in a the previously mentioned meta-analysis were mothers providing SSC to infants with low birth weight, demonstrated better family satisfaction (Conde-Agudelo et al., 2011).

Previously positive effects on psychosocial factors such as parental stress have been reported (Charpak et al., 2005). A review by Moore et al. (2012) showed that mothers who provided SSC showed less anxiety and more confidence about their abilities to take care of the infant after hospital discharge. These findings have similarities with what here have been synthesized as SSC relieving emotional suffering, feeling needed and that SSC was experienced as offering some degree of control over the situation. Furthermore, the current results point at SSC being experienced as a learning experience, which leads to an improved self-esteem.

The results also highlighted some problems experienced by the parents in a more obvious way than previously shown in individual papers. Besides reporting practical and emotional obstacles to providing SSC it was also evident that many parents were afraid of hurting their infant and uncertain of their own capacity. This knowledge is important when planning for and performing SSC-programs in health care settings.

Today, SSC is provided by parents within neonatal care worldwide, although the degree to which SSC is practiced varies (Kymre, 2014; Nyqvist et al., 2010; Olsson et al., 2012). Meta-analyses have been conducted on physiological and psychosocial outcomes (Conde-Agudelo et al., 2011; Lawn et al., 2010; McCall et al., 2010). Nevertheless, a qualitative systematic review can add other perspectives (Noyes & Hayter, 2013). For example, this review highlights the importance of support for a positive experience of providing SSC, as well as offers information of what can hamper such an experience. Such knowledge is important to take into consideration in order to further develop the SSC worldwide. Reviews have the potential to be of importance on policy and practice (Noyes & Hayter, 2013). Authorities assessing health care interventions from a broad perspective, covering medical, economic, ethical, and social aspects argue for the combination of health technology assessment (HTA) and synthesis of qualitative studies to provide decision-makers with the best possible evidence-based foundation (SBU, 2014). Mixed methods or triangulation of methodology can provide a more comprehensive picture than a single methodology (Heale & Forbes, 2013). Reviews of qualitative evidence are also regarded as important to develop theories and hypothesis (Noyes & Hayter, 2013).

The major strength of this literature review is that findings published in languages other than English have been included. This means covering large geographical and cultural areas, as well as most published research up until 2013. Two or three researchers participated in all steps (selection, appraisal, data extraction, and analysis) of this meta-research study. The use of nVivo8 facilitated the analysis phase by providing a good overview of the data, thus enabling the process of identifying patterns (*c.f*. Polit & Beck, 2012). Other authors have identified disadvantages using software programs, such as too early closure of the analysis, or the inflexibility resulting in inability to testing the reliability of categorizations (Krippendorff, 2004). However, we did not experience these disadvantages. To enable analysis all material had to be translated into English, leading to a risk of loss of nuances in the interpretation of the findings. One limitation is the uneven distribution between mothers and fathers in the included original papers. We have in our results section tried to deal with this by being faithful and write mothers when the findings relate to mothers, fathers when they relate to fathers, and parents in situations where the gender of the parent have not been clearly defined, and when the results actually refers to parents. We suggest further research on the gender-specific roles of parents in providing SSC.

Another possible limitation is that three of the original papers included report the experiences from delivery care settings while the rest reflect experiences from neonatal or KMC-settings. It was judged as best to include those three articles as they met the inclusion criteria in the searches. The findings from these papers do not render any exclusive categories; they just add some nuances in the overall identified pattern.

## Conclusion

This qualitative literature review has added scientific and systematic knowledge about parental experiences of providing SSC to their newborn infant. It constitutes a valuable complement to previous meta-analyses on physiological and psychosocial outcomes on mothers and infants, and it offers a more detailed picture than the previous meta-analyses on the topic. From an evidence-based perspective, this systematic review shows that mothers and fathers who provide SSC can experience the SSC as restorative, as well as energy-draining. However, as the mothers’ experiences previously have dominated research of the parental experiences of SSC, studies about the fathers’ experiences of providing SSC should be undertaken, in various geographical and cultural settings. Such knowledge could lead to a better understanding of the fathers’ situation within neonatal care.
